# In Vitro Techniques for Seed Potato (*Solanum tuberosum* L.) Tuber Production: A Systematic Review

**DOI:** 10.3390/plants14172777

**Published:** 2025-09-05

**Authors:** Guillermo Alexander Jácome Sarchi, Nataly Tatiana Coronel Montesdeoca, Francisca Hernández, Rafael Todos Santos Martínez

**Affiliations:** 1 Grupo de Investigación Agricultura Sostenible (GIAS), Universidad Politécnica Estatal del Carchi, Carrera de Agropecuaria, Tulcán 040102, Ecuador; guillermo.jacome@upec.edu.ec (G.A.J.S.); nataly.coronel@upec.edu.ec (N.T.C.M.); 2 Grupo de Investigación en Fruticultura y Técnicas de Producción, Instituto de Investigación e Innovación Agroalimentaria y Agroambiental (CIAGRO-UMH), Universidad Miguel Hernández, Carretera de Beniel, km 3.2, 03312 Orihuela, Alicante, Spain; francisca.hernandez@umh.es

**Keywords:** in vitro culture, micropropagation, seed tubers, microtubers, bioreactors, pathogen-free, germplasm

## Abstract

In vitro culture has become a key tool to produce seed potato (*Solanum tuberosum* L.) tubers, a crop of great global importance. This systematic review, based on the PRISMA-ScR methodology, analyzes the main biotechnological strategies used to obtain high-quality, healthy, and pathogen-free seeds, overcoming the limitations of traditional propagation methods. A comprehensive search was conducted in Scopus, Web of Science, and ScienceDirect (June 2025), prioritizing 65 experimental studies published between 2010 and 2025 in indexed journals. Techniques such as meristem culture, micropropagation, microtuber production, the use of temporary immersion bioreactor systems (TIBs), and synthetic seed generation were examined. These methodologies offer advantages such as accelerated propagation, higher yields, reduced use of agrochemicals, germplasm conservation, and economic efficiency. TIBs stand out for improving the survival and productivity of basic seed. This review is organized around four axes: applied techniques, key procedures, economic impact and sustainability, and future perspectives. This work constitutes a useful guide for optimizing seed tuber production using plant biotechnology.

## 1. Introduction

In vitro culture has emerged as a crucial technique in modern agriculture, especially in the production of seed potato tubers (*Solanum tuberosum* L.) [1]. Potato, with an annual production of 400 million tons, is positioned as one of the most important food crops globally, comparable in importance to wheat, rice, and maize [2]. Indeed, its high nutritional value makes it the third or fourth most important food crop worldwide [3,4]. Given its importance in global food security [5], the production of high-quality seed tubers using advanced techniques, such as in vitro culture, is essential to meet the growing demand [6]. This technique offers significant advantages over traditional methods by allowing rapid and efficient propagation of disease-free plants, which is crucial for ensuring crop quality and yield [7].

In vitro culture is consolidating as a biotechnological tool of great relevance for the efficient production of potato plants, overcoming the limitations of conventional methods by obtaining large quantities of uniform and pathogen-free plant material in reduced spaces and in less time [8,9]. This method is based on the micropropagation of explants, a process influenced by critical factors such as enriched Murashige and Skoog (MS) culture medium, growth regulators, aseptic conditions, pH, and optimized illumination [4,10]. Plant tissue culture has multiple applications in potato production, including efficient propagation of selected genotypes, germplasm conservation, genetic exchange, study of biotic and abiotic interactions, production of genetically modified plants, and obtaining pathogen-free planting material [11,12]. The quality of the seed tuber obtained by these in vitro techniques is a vitally important factor in the productivity and sustainability of potato production systems. The use of seed tubers free of pathogens and with high germination capacity contributes to a reduction in disease incidence, which, in turn, reduces the need for the application of agrochemicals and promotes more environmentally friendly agricultural practices [13].

The implementation of these biotechnologies can ensure consistent production of quality seed, meet farmers’ demands, and potentially reduce production costs globally [5]. The purpose of this systematic review is to provide a comprehensive and up-to-date overview of the various in vitro culture techniques employed in the production of seed potato (*Solanum tuberosum* L.) tubers, an essential biotechnological tool for mass propagation of healthy and disease-free plants [14]. In addition, the challenges inherent to these techniques, their economic impact in terms of cost and efficiency [15], and their sustainability as an alternative to conventional propagation methods, which can be conducive to pathogen accumulation [14], will be discussed. Ultimately, the purpose of this review is to elucidate the implications for future research in protocol optimization and the development of new applications in the agricultural sector. It stands as an essential guide for researchers, agronomists, and growers interested in enhancing the efficiency and quality of seed potato tuber production by taking advantage of in vitro culture techniques [16].

## 2. Literature Review

**Figure 1:**
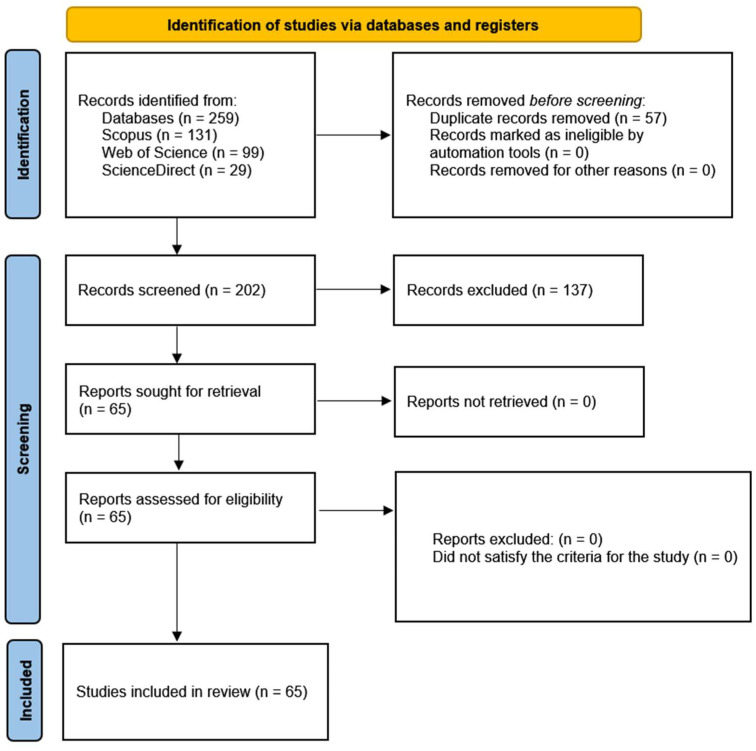
Diagram showing the selection of scientific publications. Source: [17]. This work is licensed under CC BY 4.0. To view a copy of this license, visit https://creativecommons.org/licenses/by/4.0/ (accessed on 7 July 2025).

To conduct this review, the authors adopted a research article format and a scoping review methodology, an appropriate methodological choice to provide a comprehensive and up-to-date perspective on the various in vitro culture techniques in seed potato tuber production. In accordance with the PRISMA 2020 statement [17] and the PRISMA Extension for Scoping Reviews [18], a comprehensive literature review was conducted in the Scopus, Web of Science, and ScienceDirect databases in the month of June 2025 (Figure 1). These databases are of great relevance due to their wide coverage of indexed scientific literature in the fields of biotechnology and agriculture. A comprehensive selection of keywords was made, including “micropropagation,” “in vitro propagation,” “plant tissue culture,” “potato,” “Solanum tuberosum,” “seed tubers,” and “microtubercle.” This selection of terms was meticulously designed to cover both general aspects of in vitro culture and those specific to potato and seed tubers. Priority was given to studies published in journals indexed in the Journal Citation Reports (2010 to 2025), and only research articles containing experimental design and data analysis were selected, thus ensuring the quality and scientific rigor of the included studies and allowing a more objective evaluation of the techniques and their results. The structure of this review allowed a detailed analysis of the following points: (i) in vitro seed potato tuber culture techniques, (ii) key procedures for in vitro seed potato tuber culture, (iii) economic impact and sustainability, and (iv) practical applications and future research. This systematic review was conducted in accordance with PRISMA methodological recommendations; the completed PRISMA 2020 checklist (Supplementary Materials) and standardized flow diagram are provided as Figure 1. The review protocol was not registered in PROSPERO since this registry is primarily designed for health-related systematic reviews.

## 3. In Vitro Potato Seed Tuber Cultivation Techniques

**Figure 2:**
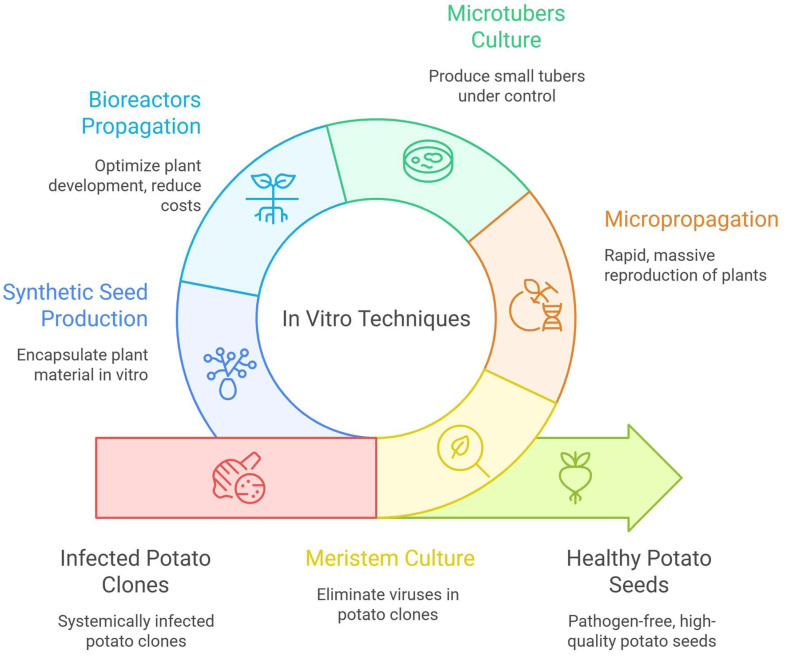
In vitro techniques to produce potato seed tubers.

In vitro culture is a fundamental tool to produce seed potato tubers free of diseases and with homogeneous characteristics. In this section, the most relevant techniques that have been used in the development of the process are precisely described (Figure 2).

### 3.1. Meristem Culture

Meristem culture is a fundamental technique for pathogen eradication. While the pioneering work of Morel and Martin (1952) demonstrated its effectiveness in dahlia [19], its successful application in potato required a combination with other techniques to ensure pathogen removal. Specifically, the protocol was optimized by coupling meristem culture with a preceding thermotherapy (heat treatment) of the mother plants, a method proven effective for cleaning infected clones [20]. This combined procedure became the standard starting point for producing clean planting material. To certify the absence of viruses in the regenerated material, subsequent diagnostic testing was essential. At the time, the ELISA (Enzyme-Linked Immunosorbent Assay) test was the mandatory confirmatory tool to verify that the propagated plant tissue was indeed virus-free [6,21]. This integrated approach forms the basis for generating healthy, pathogen-free seeds, a prerequisite for any certification program [22,23].

### 3.2. In Vitro Micropropagation

In vitro micropropagation is a key biotechnological tool for the rapid and massive reproduction of plant material. Its development as a standardized method was made possible by an understanding of the regulation of plant development by phytohormones, specifically the balance between auxins and cytokinins that controls morphogenesis, as described by Murashige (1974) in his proposal of the four stages of micropropagation [24]. Although the culture medium developed by Murashige and Skoog (1962) is now universally used, it was originally formulated for tobacco callus culture more than a decade before micropropagation became widespread [25]. Its robust mineral composition subsequently proved to be well-suited for tissue culture of a wide range of species, including potato [21,26]. A peculiarity of potato is its strong apical dominance, which inhibits the growth of lower axillary buds. To overcome this, a landmark study demonstrated that efficient micropropagation is possible by cultivating single-node explants on a hormone-free medium [27]. This method is notable because it avoids residual hormonal effects in subsequent subcultures, minimizes the risk of somaclonal variation, and generates vigorous shoots while maintaining genetic stability. Further innovation connected micropropagation with tuber production through the development of a biphasic liquid culture system [28]. This approach combines an initial shoot multiplication phase with a tuberization phase, induced simply by increasing the sucrose concentration in the hormone-free medium. This laid the groundwork for larger-scale production of microtubers, or minitubers for pre-basic seed, in bioreactors [6,29,30].

### 3.3. Microtubers Culture

The production of in vitro tubers, or “microtubers”, emerged as an efficient alternative for the propagation and storage of germplasm, with key protocols being established in the late 1970s and early 1980s [31]. Microtubers culture allows the production of small tubers under controlled conditions, ideal for the propagation of crops such as potatoes and their efficient storage. These microtubers, generated in vitro from plant tissues, have similar characteristics to field tubers and can be directly planted [10,11]. Microtuberization is a production process meticulously carried out in sterile environments, using nutrient media, preferably liquid, supported by growth regulators and specific conditions, such as photoperiod and temperature [32,33]. In addition to being healthy and pathogen-free planting material, their small size facilitates the transport and exchange of germplasm. Microtubers have a crucial application in the production of high-quality certified seeds and in genetic and molecular research, due to their easy handling and fast growth cycle. Their implementation has proven to be an efficient alternative, with significant improvements in crop survival and yield compared to other propagation methods [1,12].

### 3.4. Propagation in Bioreactors

The use of bioreactors for micropropagation in liquid media marked a significant advance towards automation, being key to the adaptation of temporary immersion bioreactor systems (TIBs) in mass production [34]. Bioreactors are highly efficient systems for the mass propagation of potato plants and microtubers. In this sense, (TIBs) stand out as an element of special relevance, given their capacity to optimize plant development through periodic immersions in liquid media [8,35]. TIBs favor gas exchange and nutrient uptake, which translates into increased shoot growth and improved root system quality [10]. These technologies have the potential to reduce production costs, ensure seed purity, and facilitate automation, consolidating as a scalable solution for the agricultural sector. In the field of agronomic research, it has been observed that microtubers obtained in certain systems have exhibited outstanding performance in basic seed production. This phenomenon is attributed to the optimization of environmental factors such as immersion frequency and the composition of the nutrient medium, which have been meticulously adapted to favor their formation and development [35,36].

### 3.5. Synthetic Seed Production

The concept of synthetic seed, based on the encapsulation of somatic embryos to simulate a true seed, was proposed in the late 1970s as an innovative technology for clonal propagation [24]. Encapsulation of plant material in vitro, such as somatic embryos or microshoots, is a rapid propagation technique with high potential and yield. The procedure is based on the generation of artificial seeds by encapsulation of somatic embryos or uninodal cuttings with root primordia in a sodium alginate solution, followed by complexation with CaCl_2_ and a dilute nutrient medium that acts as an artificial endosperm [37]. Despite the absence of an exhaustive description of its implementation in the mass production of seed tubers for the field, its efficiency and versatility position it as a promising alternative for the generation of healthy and easy-to-manage propagative material [38].

The following is a comparative summary table showing different in vitro culture techniques for potato seed tuber production (Table 1).

## 4. Key Procedure for the In Vitro Cultivation of Seed Potato Tubers

The in vitro culture procedure for seed potato tubers comprises a series of critical steps aimed at ensuring the production of disease-free planting material with homogeneous characteristics and adapted to agricultural needs. The key aspects of this process are presented below.

### 4.1. Selection and Preparation of Initial Planting Material

The production of tubers for potato planting by in vitro culture begins with the meticulous selection of plant material, a fundamental procedure to ensure healthy, pathogen-free plants with desirable genetic characteristics, such as pest resistance, high yield, and tuber quality. The choice of appropriate mother plants is essential to preserve desirable qualities in propagated plants and to prevent the introduction of pathogens, which could compromise the process. In addition, the selection of uniform material in terms of size and development facilitates more consistent management and predictable results, as evidenced in the literature references [38,39,40]. A relevant aspect to consider is the challenge posed by the accumulation of viruses in tubers over generations, which complicates their elimination by conventional methods. To address this problem, in vitro micropropagation, combined with meristem culture, has been one of the first biotechnological strategies employed, which has allowed the production of pathogen-free seeds with high sanitary potential [21,35].

The key considerations for plant material selection are as follows:

Genetic traits: The selection of mother plants with inherent disease resistance has been shown to be essential for maintaining the health of propagated plants and minimizing the risk of pathogen introduction into in vitro culture. In addition, it is critical that mother plants exhibit high yield and superior tuber quality, ensuring that these characteristics are passed on to propagated plants [26,46].

Phytosanitary quality: It is imperative to use plant material free of viruses, bacteria, and fungi to prevent contamination and guarantee the healthiness of in vitro cultures. In the field of agriculture, the use of tissue culture techniques, such as thermotherapy, chemotherapy, and electrotherapy, has proven to be effective in the production of pathogen-free planting material. This procedure results in the elimination of viruses such as potato virus Y (PVY), which is a significant advance in improving plant health [47].

In potato in vitro culture, several types of explants can be used; the most commonly used are detailed in Table 2.

The choice of explant type is influenced by several factors, such as culture strain, medium conditions, season, temperature, photoperiod, and the specific combination of plant growth regulators (PGRs) [39].

Once the initial plant material has been selected, it is imperative to carry out a rigorous disinfection process to eliminate surface contaminants, especially microorganisms, that could affect the in vitro culture [26,43]. The procedure described in this study consists of a series of fundamental steps. First, the buds or shoots are immersed in a 70% ethanol solution for approximately 30 s. Subsequently, the plant material is treated with 2% sodium hypochlorite for a period of 15 min to eliminate possible contaminants present. Subsequently, rinses with sterile distilled water are implemented for the elimination of residues of the disinfectant agent [21]. In addition, a pre-disinfection process applied to 2 cm sprouts before the main procedure is mentioned [23], together with the use of 0.1% chlorine dioxide as a sterilizing agent [56].

Once the disinfection process is complete, explants, such as nodal sections, are meticulously trimmed to an appropriate dimension before being transferred to sterile culture medium. The success of this initial phase, termed establishment, can be conditioned by a few factors, including illumination conditions [21,56].

### 4.2. Establishment and Multiplication (Micropropagation)

Micropropagation, as a relevant biotechnological technique, constitutes an essential component in the field of mass production of potato plants, guaranteeing that planting material, characterized by healthiness and absence of pathogens, is obtained [45,57]. This procedure involves the replication of plant material from explants [21]. The in vitro establishment procedure constitutes the initial phase of the protocol, in which the explant is introduced into a sterile medium. Subsequently, the multiplication phase is carried out, the objective of which is to increase the number of shoots [26].

The success of in vitro culture depends on key factors such as asepsis to prevent contamination, genotype, which influences the response to hormonal treatments, and explant type, with the nodal segment being the most effective [45,58]. In addition, the composition of the culture medium provides nutrients, carbon, and growth regulators that are essential for plant development [21,59]. Ultimately, environmental conditions, such as lighting, temperature, and gas control, optimize culture development [11,42].

#### 4.2.1. Composition of the Culture Medium and Plant Growth Regulators (PGRs)

Base medium: Murashige and Skoog (MS) medium is most frequently used as a base, since it provides essential macronutrients and micronutrients, as well as vitamins such as myo-inositol, nicotinic acid, pyridoxine, and thiamine [60,61]. Depending on the objective set, it is possible to use MS in its full concentration or reduced by half (1/2 MS) for the purpose of promoting the development of root primordia [11,42]. For solidification of the material, a binding agent is incorporated in a proportion of 0.8%. The pH of the medium should be adjusted to a range covering 5.6 to 5.8, as established in the scientific literature [58].

Carbon source: Sucrose is the most commonly used carbon source in the context of in vitro culture, because, under such conditions, photosynthesis tends to be insufficient to ensure optimal development [37]. Standard concentrations may experience variations within the range of 20 g/L (2%) to 30 g/L (3%), or according to the specific needs of the explant [42,61]. In certain circumstances, such as in the microtuberization process, higher concentrations are used, ranging from 90 g/L (9%) to 100 g/L (10%). The choice of the ideal concentration is a major factor in optimizing the development of cultured tissues [35,62].

Solidifying agent: Agar and Gelrite™ are commonly used, the concentration of which varies according to the protocol. Generally, 8.0 g/L or 7 g/L amounts of agar are used [21], while Gelrite™ is used at lower concentrations, such as 3.0 g/L, due to its higher gelling capacity [45]. The choice of agent and its concentration exert an influence on the texture and stability of the culture medium, aspects that are key for optimal explant development [11,63].

Plant growth regulators (PGRs): These elements play a primary role in the process of guiding cell development and differentiation in the plant kingdom. These substances, diverse in nature, exert control over a variety of physiological processes, thus allowing the formation and adaptation of tissues according to in vitro culture conditions [21,39,45].

- Auxins, such as 1-naphthaleneacetic acid (NAA), indole-3-acetic acid (IAA), and indole-3-butyric acid (IBA), are incorporated for the purpose of optimizing various developmental parameters, exerting a significant influence on shoot length, the number of nodes, and the number of leaves [39,64]. IBA is used in certain systems for the formation of nodes and the induction of root primordia, which favors rooting of explants [37,42].
- Cytokinins, such as 6-benzylaminopurine (BAP) and kinetin, have been shown to induce essential processes for cell development, such as cell division, growth, and differentiation, contributing significantly to tissue regeneration. BAP is essential for the establishment of tuber shoot culture and shoot proliferation [45], while kinetin is incorporated in media designed for meristems or in temporary immersion systems, where it optimizes explant response [35]. In the field of molecular and cell biology, zeatin emerges as a cytokinin of significant relevance in the context of embryogenesis. Its research has been characterized by its remarkable impact on somatic embryo formation, highlighting its importance in embryonic developmental processes [43].
- Gibberellins, in particular, gibberellic acid (GA_3_), play a crucial role in the in vitro culture process, particularly in the stem elongation phase [52]. Their combination with auxins, such as naphthaleneacetic acid (NAA), is decisive in stimulating explant growth and development [23]. A 20:1 ratio (0.2 mg/L GA_3_ and 0.01 mg/L NAA) has been shown to induce longer stems with more nodes [52]. In botany, gibberellic acid (GA_3_) is used to induce the breakdown of dormancy in potato (*Solanum tuberosum* L.) seeds [61]. Furthermore, in media designed for the proliferation of root primordia or meristems, GA_3_ has been shown to favor their regeneration and development [35].
- Other combinations: The interaction between auxins and gibberellic acid (GA_3_) is crucial to achieve efficient regeneration and adequate axillary bud growth in binodal explants. This combination optimizes the formation and elongation of vegetative structures. Regarding the differentiation of aerial parts from shoot apices, the use of a medium presenting a higher concentration of cytokinins than auxins is recommended, resulting in increased cell proliferation and shoot development in vitro culture [39].

The following are the recommended culture media for each type of explant (Table 3).

#### 4.2.2. Other Additives

In addition to the main components of the culture medium, it is possible to incorporate other additives for the purpose of optimizing in vitro development. Among these components are vitamins and amino acids, such as glycine, which play a crucial role in the cell regeneration process [11]. In scientific research, it has been observed that certain complex organic additives, such as activated carbon, can promote the growth and differentiation of explants. Furthermore, it has been shown that these additives can be incorporated into synthetic seed capsules, which is a significant advance in the field of sustainable agriculture [15]. Likewise, inositol, a type of carbohydrate, has been used to promote cell formation and development [37]. To mitigate the adverse effects of ethylene, silver nitrate (AgNO_3_) has been employed, which has improved explant response [26]; similarly, the combination of AgNO_3_ (2 mg/L) with NAA (0.1 mg/L) optimizes rooting and microtuberization [69]. In this regard, some fungicides, such as thiophanate methyl (TM), can be incorporated into the culture medium to control contamination in synthetic seed cultures and ensure optimal conditions for their development [42].

#### 4.2.3. Environmental Conditions

Environmental conditions play a crucial role in in vitro culture, exerting a significant influence on explant growth and development. The hypothesis of this study focuses on the relevance of illumination in the process of morphogenesis and microtuberization. The observation of a photoperiod of 16 h of light and 8 h of darkness is proposed as a fundamental premise, as a key factor for ensuring optimal photosynthetic activity [61]. Several studies have shown that the exposure of plants to total white fluorescent light, in combination with 12 h of natural light and 12 h of darkness, favors their vertical growth. In addition, the quality of light, and, in particular, the ratio between red and blue light, has been found to exert a significant impact on plant morphology [21]. Regarding temperature, it has been established that the optimal range for the balanced development of organisms is between 21 and 25 °C [11,26]. On the other hand, gas exchange is crucial since the accumulation of ethylene in closed containers can inhibit shoot growth. To address this issue, temporary immersion bioreactor systems (TIBs) are implemented, which facilitate the renewal of gases, optimizing the development of explants under controlled conditions [10].

### 4.3. Induction and Production of Microtubers In Vitro

The implementation of in vitro tuberization facilitates the expeditious propagation of pathogen-free potatoes through the formation of microtubers, which are small tubers obtained through tissue culture. These procedures contribute to the conservation, distribution, and production of pathogen-free seeds [26]. In addition, these procedures have been shown to contribute to the preservation of germplasm and the development of transgenic plants [21]. They are also used in the process of seed multiplication and germplasm distribution, thus ensuring crop health [10].

The process of microtuber production under in vitro conditions is characterized by the presence of two main phases. First, plant proliferation is carried out, in which explants generate new shoots through optimized culture conditions, promoting their vegetative growth. In the second stage, the development of microtubers is promoted by regulating factors such as sucrose concentration, photoperiod, and the presence of growth regulators, with the aim of inducing tuber formation [62].

Several factors influence in vitro microtuberization, including culture medium, growth regulators (phytohormones), lighting conditions, explant type, culture systems, and yield [21].

#### 4.3.1. Culture Medium

Sucrose is a vital component for the development and formation of the cell structure of potato (*Solanum tuberosum* L.) under in vitro culture conditions [21]. The concentration of the substance in question undergoes variations depending on the stage of culture, ranging from a minimum of 10 g/L, corresponding to the period of plant proliferation, to a maximum of 100 g/L, associated with the formation of microtubers. Numerous studies have shown that a concentration of 90 g/L in a hormone-free medium favors the production of microtubers in temporary immersion systems [37,62]. In the field of synthetic seeds, the sugar present in artificial endosperm solutions has been shown to improve root length, which provides external energy for plant development [42].

Monopotassium phosphate emerges as a highly relevant component in the culture medium, exerting a significant influence on the production of large-sized microtubers. It has been observed that a concentration of 510 mg/L significantly increases the number of microtubers larger than 3 g, compared to the standard concentration of 170 mg/L. This adjustment in phosphorus availability has the potential to optimize the yield and quality of microtubers in the context of in vitro culture [62].

#### 4.3.2. Plant Growth Regulators (PGRs)

Plant growth regulators (PGRs) are essential in the in vitro tuberization process. A protocol combining 6-benzylaminopurine, chlorocholine chloride, and sucrose has been described to induce the process [26]. Regarding microtuber production, the combination of 100 g/L sucrose, 0.75 mg/L gibberellic acid (GA_3_), and 0.30 mg/L BAP has been shown to be effective, with remarkably high production of microtubers [23]. Benzyladenine (BA) is a compound used in various contexts, such as in the medium for microtubule production in culture bag systems, where its concentration can reach 2 mg/L [62]. In the process of optimizing a medium for microtubule production in a temporary immersion system (SETIS^TM^), a concentration of 2 mg/L kinetin, 0.5 mg/L IBA, and 0.5 mg/L GA3 was used. However, the medium (4.43 g/L MS with vitamins) without hormones with 3% sucrose proved to be the most effective for microtubule production [35]. It is imperative to consider that the use of PGRs can prolong regeneration time and affect shoot, node, and root formation under suboptimal conditions, which influences healthy seed potato production [39].

#### 4.3.3. Lighting Conditions

Light emerges as a paramount factor in the context of in vitro potato (*Solanum tuberosum* L.) culture since it plays a crucial role in the regulation of cell growth, morphogenesis, and microtuberization [14,21]. The photoperiod exerts a significant influence on each stage of the process, with a period of 16 h of light exposure and 8 h of darkness being recommended for the tuberization phase. However, some protocols propose continuous darkness as a strategy for microtuber production. Variations in the number of minitubers have been observed, with increases in certain varieties under 18 h of light and improvements in dry weight in others under 12 h of light [62,68,70]. Light quality influences the morphology of microtubers, with red light being favorable for their formation, while blue light boosts their growth. The combination of white light modifies these effects. The strategic application of different light conditions at each stage optimizes microtubule production and storage [33].

#### 4.3.4. Type of Explant

Explants are essential for the establishment of potato in vitro culture, either for multiplication or tuberization purposes [21]. Among the most used are nodal segments and single-node cuttings, which have shown greater efficiency in the formation of microtubers compared to double-node cuttings [70]. Likewise, tuber and bud sprouts are used, which demand meticulous preparation, particularly regarding disinfection and light conditions, to ensure their optimal development [11,21]. Furthermore, it has been shown that apical meristems play a fundamental role in obtaining virus-free material, as they include the apical cone and leaf primordia in their structure. The appropriate choice of explant and its pretreatment are essential to optimize the yield and guarantee the quality of the in vitro culture [37].

#### 4.3.5. Culture Systems

The implementation of in vitro tuberization can be carried out in two modes: in a solidified medium with agar or in a liquid medium. A liquid medium facilitates large-scale and automated production with reduced labor costs, since changing the medium to move from the proliferation stage to the microtubule production stage is simpler in liquid [62].

Bioreactors have been employed for the mass production of microtubers, standing out for their efficiency in performance, handling, and costs. Among the various techniques employed, temporary immersion bioreactor systems (TIBs) have demonstrated remarkable efficiency by facilitating gas renewal and ensuring continuous contact with the nutrient medium, which promotes growth and development of the sprouts [10,36]. Compared to liquid culture, bioreactors have the potential to generate higher yields and reduce hyperhydricity [26]. The production of microtubers per bioreactor exhibits variations, with reported ranges ranging from 129 to 491, and their application has been shown to have a beneficial effect on plant development [10].

A system to produce microtubers in sterile plastic culture bags has been implemented, where microtubers undergo development from the surface of the medium to the top of the bags. Aeration of the gas phase inside the bags is essential for explant growth, resembling medium control in other systems. Its design facilitates a more accessible regulation of internal conditions, which positions it as a low-cost and accessible alternative for efficient microtubule production [62].

#### 4.3.6. Yield

It is evident that microtubers produced in vitro, especially those of larger size (greater than 0.3 g or 3 g), present advantages for practical use in potato (*Solanum tuberosum* L.) cultivation. In the field planting process, these larger tubers tend to give rise to plants with a greater number of stems and a greater number of tubers per plant, resulting in a higher yield and a higher harvest rate. In addition, it has been observed that larger microtubers exhibit superior adaptability to field conditions compared to smaller ones, resulting in higher development and productivity [62].

A comprehensive evaluation of yield and basic seed characteristics was carried out on Peruvian native varieties grown from microtubers under greenhouse conditions. The results obtained in the study indicate that tubers generated from microtubers exhibit outstanding performance, outperforming those obtained from cuttings. These findings suggest that microtubers possess a significant advantage in the efficient production of basic seed [10].

The procedure of obtaining basic seed from microtubers was carried out over a period of five months, constituting a strategy to ensure constant production and meet the needs of farmers. This method not only ensures sustainable production but also significantly reduces production costs [10].

The microtuber production procedure constitutes the starting point of the *Solanum tuberosum* seed cycle, beginning with the formation of minitubers (G0) from acclimatized vitroplants. These G0 minitubers multiply in successive generations, giving rise to seeds of G1, G2, and so on, ensuring a staggered and efficient production for potato (*Solanum tuberosum* L.) cultivation [30].

### 4.4. Hardening and Acclimatization

Once seedlings have been multiplied or microtubers have formed in vitro, the next crucial step is the transition from in vitro to ex vitro conditions, known as hardening or acclimatization [11,71]. This process involves the gradual adaptation of plants from an inert, high-humidity environment, typical of in vitro culture, to ex vitro conditions [72]. The primary objective of hardening is to ensure that plants possess the ability to survive, establish, and develop successfully in an environment external to the regulated laboratory environment [41,72].

The plant material used in this period comprises mainly rooted seedlings or microtubers produced in vitro [21,41]. The procedure involves growing plants in soil or various substrates in controlled environments, such as greenhouses (glasshouses), tunnels, or grow houses [30,71]. The use of beds with a 10 cm high substrate composed of a 2:1 m^3^ ratio of black soil and sawdust has been described [10]. Similarly, high survival rates of 90% were achieved for the Kufri Chipsona-1 variety with coconut fiber, perlite, and soil substrates in a 2:1:1 ratio [65] and 100% rooting in the Kufri Sangam variety in a sterile substrate composed of soil, sand, and manure in a 1:1:1 ratio [73].

The successful adaptation of plants in vitro is closely linked to the development of robust root and shoot systems [72]. It has been observed that plants obtained under certain in vitro conditions (such as partial natural lighting) can show faster adaptation to the field [74]. The implementation of a high-quality root system prior to transplanting, using temporary immersion bioreactors (TIBs), can facilitate photosynthetic adaptation of plants and promote accelerated post-transplant growth [10]. Similarly, obtaining complete plants with a well-developed root system in vitro is also important [35].

Plant survival is a key indicator of effective acclimatization [72]. Microtubers obtained from TIBs present the capacity to increase the survival rate of plants, with percentages higher than 80%, as evidenced in the Peruanita variety, where 89.04% was reached. In addition, plant height at 30 days ex vitro is a relevant indicator of propagule quality. According to some studies, a positive impact on plant development and significantly greater average heights have been observed [10].

During the acclimation process, viability can be influenced by various factors, such as the addition of substances to the medium or substrate. For example, melanin, at a concentration of 0.028%, has been shown to improve the viability of strains such as Nevsky (96%) and Impala (89%) [72]. The quality of in vitro material prior to acclimatization is influenced by several factors, such as the composition of the culture medium (hormones, sucrose) [35] and the culture conditions (photoperiod, light) [21]. Once acclimatized, the microplants are ready to be used as starting material for subsequent steps, such as the production of minitubers. Acclimatization is an essential step prior to transplanting the seedlings to greenhouses, net houses, or even directly to the final field [21].

### 4.5. Ex Vitro Seed Tuber Production (Minitubers)

Once the hardening and acclimatization phase is completed, the micropropagated plants or microtubers obtained in vitro advance to the ex vitro seed tuber production stage, called minitubers [30,71]. This procedure constitutes an essential component in seed potato production programs, with the purpose of obtaining seed material that is healthy and of superior quality [1,26].

There are several methods for ex vitro growth and tuberization of plants:

- Soil or substrates: Seedlings can be transplanted directly into soil or different types of substrates [71]. It has been documented that the use of mineral substrates, such as coarse sand, optimizes the acclimatization process and increases the survival rate in the field [26]. Soil-less substrate configurations, growing beds, or containers are also implemented [1].
- Soilless systems: Technologies such as hydroponics, semi-hydroponics, and aeroponics have been used to optimize growth [1]. Aeroponics is considered a modern and efficient technique for minituber production, providing a soilless environment where roots and subway stems develop in a dark chamber. In this system, water and nutrients are supplied by a sprayed solution, which facilitates the formation of minitubers in subway stolons [11].

Minitubers are potato tuberous structures obtained ex vitro. The size of the sample under study presents an intrinsic variability, with reported diameters ranging from 8 to 28 mm, or in a range of 4 to 12 mm [1,30]. Under greenhouse conditions and using in vitro microtubers, there is evidence of a greater generation of smaller tubers, thus facilitating their use in seed production programs due to the simplification involved in their handling and transport [10]. The yield in the production of minitubers is influenced by several factors. In conventional substrate systems, production tends to be limited, with an average of two to five tubers per microplant [26,41]. However, advanced technologies, such as aeroponics, have proven to be more efficient, achieving between 30 and 170 minitubers per plant in each production cycle [11].

The genotypes and types of planting material also play a key role in the quantity, size, and total yield of minitubers. In vitro cultures of varieties such as Kennebec and Agria have shown a substantial increase in minituber production under aeroponics conditions, with values of 53.8 and 54.5, respectively [11]. In addition, environmental factors, such as photoperiod, temperature, and photosynthetic capacity, affect tuber distribution and growth [70].

The quality of the seedlings at the end of the in vitro stage is decisive in their ex vitro adaptation and acclimatization. Those with a robust root system and optimal chloroplast development exhibit a greater photosynthetic capacity after transplanting, which can result in the formation of larger and heavier tubers [70]. In addition, the use of microtubers derived from temporary immersion bioreactor systems (TIBs) has been shown to increase survival and yield of tubers intended for basic seed production [10].

Minitubers, as a pathogen-free starting material, are employed in subsequent stages of the seed potato production program [26]. Seed cultivation is carried out in fields intended for this purpose, with the aim of increasing the volume of planting material, which includes basic seed and other categories [1]. In this sense, local seed production by laboratory vitropropagation techniques, followed by successive field multiplications, emerges as a more competitive alternative compared to schemes that rely on imported minitubers [30].

### 4.6. Quality Control and Sanitation

Ex vitro production is carried out in controlled environments, such as greenhouses or greenhouses equipped with anti-aphid netting, to protect the plants from pests and diseases. In such spaces, multiple characteristics are evaluated to guarantee the quality of the basic seed, including survival, plant height at 30 and 80 days, number of tubers per plant, diameter, weight, size, and skin color of the tubers. Yield, quantified in kilograms per area, is a primary indicator of productivity. Recent studies have shown that minitubers derived from microtubers exhibit superior yield compared to cuttings [10].

In advanced systems such as aeroponics, used for asexual propagation, the precise control of environmental parameters, such as temperature, humidity, light, and CO_2_ concentration, together with the regulation of the nutrient solution (pH, electrical conductivity, nutrient concentration, and dissolved oxygen), is essential for the success of the crop. Maintaining an optimum pH between 5.7 and 6.3 facilitates nutrient absorption and prevents growth problems, while a balanced electrical conductivity prevents stress due to excess salt or nutrient deficiencies. These controls are essential for ensuring the health and proper development of plants in protected environments [37].

Sanitation is an important aspect of seed tuber production, as historically, it has lacked strict regulations, which can make crops vulnerable to viral and bacterial diseases, as well as to pest attacks [10]. The implementation of biotechnology in the production chain, such as micropropagation, guarantees propagation material with high phytosanitary quality [26]. In particular, in vitro culture using apical meristems is essential for establishing a healthy variety bank and guaranteeing the absence of viral infections in plants [35]. Throughout the process, including the ex vitro phase, the aim is to obtain tubers that meet quality standards, with a lower incidence of diseases and greater tolerance to adverse conditions [30]. In this sense, the production of basic seeds from microtubers obtained in temporary immersion bioreactor systems (TIBs) under controlled conditions is presented as an effective alternative to guarantee a constant supply of healthy seed [10].

**Figure 3:**
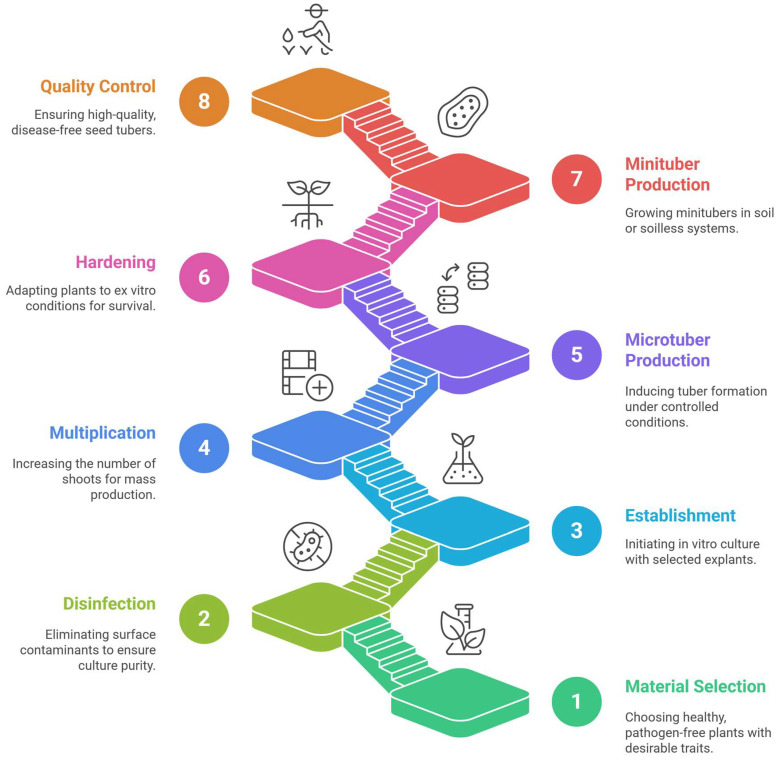
Steps to produce seed potato tubers.

The basic steps for obtaining seed potato tubers are detailed below (Figure 3).

## 5. Economic Impact and Sustainability

The implementation of in vitro culture techniques for seed potato tuber production has a significant impact on the economics and sustainability of agricultural systems by offering solutions to the limitations of conventional propagation [21,26]. Traditional multiplication of *Solanum tuberosum* by tubers carries a high risk of pathogen accumulation and dispersal, which compromises the productivity of future crops [26]. In regions such as the Andes, where traditional production often lacks regulations and standardized techniques, vulnerability to viral and bacterial diseases, as well as pests, generates cost overruns, yield losses, and a decrease in crop quality [10,45]. The native potato, an underutilized tuber, is exposed to a high risk of spreading pathologies through the tubers used for planting, resulting in reduced agricultural yields [45].

In contrast, in vitro production of potato seed tubers with high genetic and phytosanitary standards emerges as an economically viable option [10]. Despite being an essential component for producing clean and rapid planting material, commercializing in vitro potato seeds is risky due to their high costs and operational risks, requiring impeccable management to be profitable [21]. The use of propagative material of high phytosanitary quality contributes to a reduction in pest and disease incidence at later stages, which can reduce the need for pesticides and, therefore, favor more sustainable practices [1].

Temporary immersion bioreactor systems (TIBs) optimize potato (*Solanum tuberosum* L.) micropropagation by improving shoot development, which is achieved through constant gas exchange and contact with the nutrient medium. The microtubers obtained in TIBs present outstanding characteristics in terms of survival, number, diameter, and weight, particularly in varieties such as “Peruanita” and “Amarilla Tumbay”. In contrast, the Diacol Capiro variety achieved a survival rate of 80% and higher production per plant. From an economic perspective, microtubers generated in TIBs have shown superior yields in basic seed, reaching 26.89 ± 0.483 kg per 14.05 m^2^, thanks to better root development that favors photosynthetic adaptation and accelerated growth. In agriculture, production over a five-month period guarantees a constant supply. This circumstance leads to a reduction in costs and facilitates access to farmers [10].

In terms of sustainability, soil-free technologies, such as aeroponics, complement in vitro techniques by allowing the production of seed tubers and avoiding contact with soil pathogens. These systems not only facilitate cleaning but also allow staggered harvests, standardize tubers, and guarantee high phytosanitary quality and a higher multiplication rate [26]. Aeroponics emerges as a viable alternative in developing countries such as Ecuador [18]. Soilless systems, such as aeroponics, are considered rapid propagation techniques with high potential and yield. Maintaining precise control of parameters such as electrical conductivity (EC) in such systems is vital for optimal growth and plant health, resulting in greater sustainability [37]. Nevertheless, their economic sustainability is precarious; these systems are energy-dependent and require precise management. Interruptions in power or failures in environmental control can lead to catastrophic crop loss, representing a significant financial risk that has deterred large-scale commercial investment [75].

Biofertilizers, as soil improvement agents, have been shown to promote sustainability and generate a positive economic impact on agricultural systems. These biofertilizers have been designed to improve nutrient uptake, growth, and yield of crops, in addition to increasing their resistance to stress. Vermicompost has proven to be highly effective, and its combination with the “Spunta” variety and microtubers generated the highest seed tuber yield (173.12 g). In this sense, mycorrhization emerges as a complementary strategy that can optimize quality and economic yield in potato micropropagation. This approach reduces dependence on chemical fertilizers and promotes more sustainable agricultural practices [1].

In addition to optimizing seed production, in vitro techniques are essential for the conservation of genetic resources, ensuring the availability of varieties and strengthening long-term crop resilience through cryopreservation and tissue culture [26,45]. While economic evaluations and initial costs must be considered, the available evidence indicates that biotechnology applied to basic seed production is feasible for native varieties. Vitropropagation constitutes an effective strategy for food security and agricultural development by reducing dependence on imported seed and improving local production [37].

In vitro propagation constitutes an effective strategy for food security primarily in controlled, subsidized, or high-value scenarios. For broader commercial viability, future innovation must focus not only on improving technical efficiency but also on drastically reducing production costs and simplifying operational logistics to overcome the historical barriers that have plagued the industry [75].

## 6. Practical Applications and Future Research

In vitro culture techniques and potato micropropagation offer key applications in agricultural production and present opportunities for future research. Among the main benefits derived from their implementation are the production of high genetic and phytosanitary quality seeds, free of pathogens, which translates into a reduction in pest incidence and a minimization of pesticide use [26,45]. The formation of microtubers in systems such as temporary immersion bioreactor systems (TIBs) has demonstrated higher yield and survival, outperforming cuttings at the basic seed stage. In addition, their application in native varieties helps to ensure sustainable production in regions where traditional propagation is vulnerable to diseases [10,23]. Soilless technologies, such as aeroponics and hydroponics, have proven effective in improving crop health and optimizing seed tuber production. Simultaneously, germplasm conservation through cryopreservation protects potato genetic diversity, while biotechnology contributes to crop improvement, strengthening food security, and agricultural efficiency [29].

Despite the progress made in the field of potato micropropagation, there are still areas that require further exploration and rigorous study. It is crucial to develop specific breeding protocols adapted to different regions and environmental conditions, as well as to conduct detailed economic evaluations to determine the commercial viability of rapid propagation systems [23]. The implementation of renewable energies has the potential to reduce large-scale production costs. On the other hand, genetic improvement aims to produce more stress-resistant plants [37]. Also, techniques for the creation of key phytochemicals and the development of smart monitoring systems using IoT sensors need to be explored, especially in aeroponics, to optimize parameters such as survival rate and root development. Research into specific nutrient solutions for soilless systems is essential, as is the evaluation of new quality parameters in basic seed. Ultimately, overcoming the technical constraints inherent to tissue culture, as well as optimizing the formulation of the basal medium, will be essential to enhance propagation efficiency, including the improvement of synthetic seeds in aspects related to maturation and storage [10].

## 7. Conclusions

Micropropagation and associated in vitro techniques hold significant technical potential for producing high-quality potato seed tubers, offering advantages in speed, multiplication rate, and phytosanitary status over conventional methods. However, this review concludes that the historical trajectory of commercial application reveals a stark contrast to this technical promise. The failure of numerous ventures dedicated to large-scale in vitro seed potato production underscores the profound economic and logistical challenges inherent in scaling laboratory protocols. High infrastructure costs, contamination risks at an industrial level, and complex logistics have consistently proven to be major barriers to commercial viability.

Consequently, while methodologies like microtuber production in temporary immersion bioreactor systems (TIBs) demonstrate high efficiency in research settings, their designation as the “most promising strategy for large-scale seed production” requires heavy qualification. Their promise is likely confined to specific, high-value niches, such as the rapid multiplication of elite germplasm, the production of pre-basic seed, or specialized markets for native varieties, rather than serving as a blanket solution for the entire seed potato industry.

The future of these technologies does not lie solely in further technical optimization but in developing integrated, cost-effective, and resilient business models. The integration of TIBs with soilless systems such as aeroponic systems and the Internet of Things (IoT) represents a compelling pathway only if it directly addresses the cited challenges of cost reduction and operational simplicity. Therefore, future research must pivot from purely agronomic goals to include rigorous socio-economic studies, detailed life-cycle assessments, and pilot projects that validate scalability and profitability under real-world conditions. Ultimately, the sustainable contribution of in vitro techniques to global food security is contingent not on their technical perfection in the lab but on their ability to overcome the formidable commercial hurdles that have defined their history.

## Figures and Tables

**Table 1:** Comparison of in vitro culture techniques for seed potato tubers.

Technique	
Meristem culture; References [21,22,26,35,39,40]	Description This technique involves isolating 0.1–0.3 mm meristematic apices with leaf primordia and cultivating them under sterile conditions using nutrient media such as MS. Its main goal is to regenerate virus-free plants while maintaining the genetic uniformity of the original clone.
	Primary use Virus elimination of infected potato clones. Production of healthy and pathogen-free seed.
	Source material Apical meristems, apices, or buds are isolated from tuber shoots or from plants grown under controlled conditions. Single-node explants can also be used.
	Conditions/Key Means In vitro culture is performed in MS medium with salts, vitamins, and sucrose (20–30 g/L, up to 80 g/L for tuberization), solidified with agar or Gelrite. Growth regulators, such as kinetin, GA_3_, IAA, BAP, IBA, jasmonic acid or daminozide, and, optionally, ribavirin, as an antiviral, are added. The pH is adjusted between 5.7 and 6.0, with temperatures of 21–26 °C. Light is essential, applying a 16/8 h photoperiod (light/dark) or short day (8/16 h) with fluorescent illumination.
	Advantages First biotechnological approach to eliminate viruses. Obtaining healthy clones. Provides initial material of high health.
	Disadvantages A laborious technique with low initial yield. It requires high specialization and strict aseptic conditions.
In vitro micropropagation; References [6,21,26,29,41]	Description A rapid multiplication method that uses plant tissue fragments (such as nodal segments or shoots) cultivated in artificial media under sterile conditions. It enables the large-scale production of healthy and uniform plants, essential for certified seed production.
	Primary use Mass production of plants allows for obtaining healthy and uniform planting material, essential for certified seed. It also facilitates the production of microtubers, improving efficiency and eliminating pathogens.
	Source material The source materials include nodal segments, cauline apices, apical buds, tuber shoots and discs, callus, leaves, stems, and axillary buds. Botanical seeds (TPS) and virus-free tubers can also be used. The seedlings obtained are used for multiplication by nodal cuttings.
	Conditions/Key Means The artificial culture medium, commonly MS or 1/2 MS, is supplemented with sucrose, gelling agents (agar or Gelrite), and growth regulators, such as BAP, kinetin, GA_3_, NAA, IAA, and IBA, as well as supplements, such as myo-inositol, vitamins, activated charcoal, and fungicides. Strict asepsis is maintained, with the pH adjusted between 5.7 and 5.8, a controlled temperature (20–27 °C), and day/night cycles. Lighting is provided by fluorescent, LED, or SON-T/HPI-T lamps, with specific intensities and a usual photoperiod of 16 h of light. Ventilation and containers vary according to the type of culture.
	Advantages Alternative for mass production of plants. Rapid multiplication of healthy clones favors the efficient multiplication of microtubers, optimizing costs and improving germplasm conservation.
	Disadvantages Risk of somaclonal variability. Requires specialized infrastructure and rigorous control of environmental conditions.
Microtubers culture; References [10,11,21,29,33,34]	Description A process that induces the formation of small potato tubers under in vitro sterile conditions, simulating field-like environments. It involves shoot proliferation followed by tuberization and is useful for basic seed production and germplasm conservation.
	Primary use Basic and certified seed production. Facilitates germplasm conservation, genetic, and molecular research. It serves as a basis for the production of minitubers in aeroponic systems. It also has the potential to generate artificial seeds from somatic embryos.
	Source material Tuber shoots, nodal sections of etiolated shoots, meristematic apices, and leaf explants can be used.
	Conditions/Key Means It uses nutrient media such as Murashige and Skoog (MS), with sucrose up to 9% for tuberization. Gelling agents, such as agar and plant growth regulators (PGRs), such as BAP, auxins (NAA, IAA, IBA), gibberellins (GA_3_), kinetin, Zeatin, 2,4-D, and TDZ, are used, adjusting their proportions according to the objective. Liquid media are preferred for large-scale production, although solid media are also used. Factors such as light (photoperiod of 16 h light or continuous darkness for tuberization), temperature (18 °C for tuberization, 25 °C for initial culture, 21 °C in controlled room, 20 °C for pre-browning), and humidity influence development. Efficiency is improved with temporary immersion bioreactor systems (TIBs) and aerated bags. Nitrogen, potassium phosphate, and activated carbon adjustments optimize tuberization.
	Advantages Alternative for the mass production of plants. Rapid multiplication of healthy clones favors the efficient multiplication of microtubers, optimizing costs and improving germplasm conservation.
	Disadvantages It requires precise adjustments to tuberization conditions. Production can be limited by environmental and physiological factors.
Propagation in bioreactors (temporary immersion systems—TIBs); References [5,42,43,44]	Description An automated system that uses temporary immersion bioreactor systems to cultivate plant material in liquid media, enhancing gas exchange and culture efficiency. It is designed for the mass production of microtubers and cuttings for basic seed generation.
	Primary use Micropropagation and mass production of plants and, specifically, of potato microtubers and cuttings. They are an alternative to produce basic seed (basic category or G0).
	Source material It is initiated from propagated material, such as single-node explants, shoots, microtubers, or cuttings.
	Conditions/Key Means Temporary immersion bioreactor systems (TIBs), such as SETIS™, optimize in vitro culture by periodically immersing plant material in a liquid medium, which improves gas exchange. Their effectiveness depends on factors such as the frequency and duration of immersion cycles (every 3 h for 2 min), the composition of the medium (modified MS with 9% sucrose, low nitrogen concentration, and growth regulators, such as kinetin, GA, and IBA), and the environmental conditions, which include specific temperatures (22 °C for growth and 18 °C for microtuberization) and appropriate photoperiods (16 h of light for growth and total darkness for microtuberization).
	Advantages They offer high yield efficiency, favoring the formation of more tubers than solid media, with greater size and weight. In addition, tubers derived from microtubers in TIBs stand out for their superior performance in the production of basic seed, compared to those obtained from cuttings.
	Disadvantages High equipment costs. Requires specialized technical management and constant monitoring.
Synthetic seed production; References [26,42,43,45]	Description A technique that encapsulates potato somatic embryos in calcium alginate matrices, derived from cell cultures. It facilitates the regeneration of complete plants and allows for efficient handling and transport of pathogen-free artificial seeds.
	Primary use Production of artificial seed potato from somatic embryos.
	Source material Somatic embryos, which can be obtained from explants such as leaves or cell cultures in suspension. Uninodal cuttings (3–4 mm) with root primordia.
	Conditions/Key Means Callus induction is performed from leaves grown on MS medium with 10 mg/L 2,4-D and TDZ, promoting high callus formation. Somatic embryogenesis is carried out on solid medium or in suspension, using combinations of regulators such as zeatin and GA_3_ (0.1 mg/L each) or BAP and GA_3_ (2.5 and 5 mg/L, respectively) to enhance development. Embryos obtained in suspension are encapsulated in 3.5% calcium or sodium alginate, with charcoal and fungicide, and supplemented with 1.1% CaCl_2_ in medium with artificial endosperm (1/2 MS).
	Advantages Mass production of somatic embryos allows for the generation of artificial seeds, which can regenerate plants and provide virus-free material. In addition, synthetic seeds are easy to handle and transport.
	Disadvantages Risk of somaclonal variation. Low rate of embryo conversion into viable plants. Technique still under development for commercial use.

**Table 2:** Comparison of explants used in potato in vitro culture.

Type of Explant	Characteristics	Main Use	References
Apical meristems	- Size: 0.1-0.2 mm (apical dome + 1–2 leaf primordia). - No vascular connections. - Regeneration rate: 85–95%.	- Virus-free plant production. - Germplasm conservation and cryopreservation.	[21,48,49]
Activated axillary buds	- Size: 2–3 mm (leaf axil-derived). - Pre-formed meristems. - Shoot induction rate: 80–90%.	- Rapid clonal multiplication. - Primary explant for mass propagation.	[21,50]
Nodal segments (with inhibited axillary buds)	- Size: 3–4 mm (containing ≥ 1 node). - High axillary shoot potential. - Propagation success: 90–95%.	- In vitro multiplication. - Rooting pre-acclimatization.	[27,51,52,53]
Tuber sprouts	- Size: 3–5 mm (surface-sterilized). - Vigor depends on mother tuber physiology - Establishment rate: 70–85%.	- Direct in vitro establishment. - Certified seed potato production.	[10,54,55]

**Table 3:** Recommended culture media for each explant type.

Type of Explant	Characteristics	References
Apical meristems	MS + 30 g/L sucrose (3% MS)	[35,49,65]
Axillary buds	MS + 1 mg/L BAP + 0.5 mg/L GA_3_	[66]
Nodal segments	MS + 5 mg/L BAP + 80 g/L sucrose (8% MS) + 0.75 mg/L GA_3_	[23,67]
Stem segments	MS + 1 mg/L BAP + 0.5 mg/L NAA	[68]
Tuber sprouts	MS + 2 mg/L BAP + 1 mg/L NAA + 60 g/L sucrose (6% MS)	[55,66]

## Supplementary Materials

The following supporting information can be downloaded at: https://www.mdpi.com/article/10.3390/plants14172777/s1, PRISMA 2020 checklist.

## Abbreviations

The following abbreviations are used in this manuscript:

2,4-D	2,4-Dichlorophenoxyacetic acid
AgNO_3_	Silver thiosulfate
BA	Benzyladenine
BAP	6-Benzylaminopurine
TIBs	Temporary immersion bioreactor systems
CaCl_2_	Calcium chloride
EC	Electrical conductivity
G0	Minitubers obtained from acclimatized in vitro plants
GA_3_	Gibberellic acid
HPI-T	Metal halide tubular lamp
IAA	Indole-3-acetic acid
IBA	Indole-3-butyric acid
LED	Light emitting diode
IoT	Internet of Things
MS	Murashige and Skoog
NAA	Naphthaleneacetic acid
PGRs	Plant growth regulators
PVY	Potato virus Y
SETIS™	A type of temporary immersion bioreactor
SON-T	High pressure sodium tubular lamp
TDZ	Thidiazuron
TM	Thiophanate methyl
TPS	Botanical seeds
